# Lichens as Bioindicators of Global Change Drivers

**DOI:** 10.3390/jof10010046

**Published:** 2024-01-05

**Authors:** Lourdes Morillas

**Affiliations:** Departamento de Biología Vegetal y Ecología, Facultad de Biología, Universidad de Sevilla, Profesor García González s/n, 41012 Sevilla, Spain; lmorillas@us.es

In recent decades, the scientific community has put the spotlight on the severe impacts that environmental stressors are producing on ecosystem functioning worldwide [1]. A broad range of interconnected stressors, such as climate change, pollution, change of land use, or biodiversity loss, also known as “global change drivers”, are collectively contributing to significant large-scale environmental impacts. These alterations pose a significant threat to lichens, known as one of the most sensitive organisms to environmental changes [2] due to their strong physiological connection to atmospheric humidity. Indeed, these poikilohydric organisms lack mechanisms to regulate water and nutrient levels [3], making them valuable ecological indicators. Their sensitivity makes them instrumental in modeling and predicting the responses of less sensitive organisms within the ecosystem [4,5]. Beyond serving as meaningful ecological models, both epiphytic and soil lichens can have a large impact on forest dynamics such as regulating nutrients and water cycling [6,7]. For example, soil lichens exert an important influence on crucial underground dynamics [8], whereas epiphytic lichens improve water use by plants as a result of their ability to regulate canopy environments [6]. Consequently, shifts in lichen communities could profoundly impact other essential forest processes. In this general context, this Special Issue, entitled “Lichens as Bioindicators of Global Change Drivers”, was launched with the purpose of advancing our understanding of how lichens can contribute to unraveling the potential effects of global change drivers, not only on lichen communities but also on key ecosystem processes derived from the influence of these organisms. The goal of this Special Issue was to provide an opportunity to integrate studies from different environmental stressors, lichen types, and geographic locations with the aim of capturing the complex responses of these organisms to the current environmental changes.

In the present Special Issue, researchers in this relevant subject have contributed five original articles to expand our understanding of the potential role of lichens in monitoring the effects of global environmental changes. Four of them were performed in three European countries, whereas only one study was performed in the USA. Out of these five studies, two of them dealt with epiphytic lichens, two with soil lichens, and only one study integrated an analysis of lichens growing on different surfaces. Changes in land use are increasingly leading natural habitats to be replaced by livestock, agriculture, and plantations of exotic species, which has an acute detrimental effect on tree species diversity. González-Montelongo and Pérez-Vargas [9] explored the host preferences for epiphytic lichens in the laurel forest of Macaronesia (i.e., the Canary Islands) by analyzing the lichen communities on the four most prevalent trees in these forests. They found a recurring and consistent pattern in diversity, richness, and lichen composition, aligned with the tree functional traits that were studied. These results underscore the relevance of the natural diversity of tree species in the laurel forest and stress the dangers involved in forest fragmentation.

Long-term studies are crucial to determine the progress of the effects of environmental factors on lichens in an integrative manner. Moya et al. [10] studied the evolution of the state of epiphytic lichen communities in forests in seven sampling points within a biomonitoring network in the eastern Iberian Peninsula. The lichen community was first assessed in 1997 and then reassessed in 2022 using the same methodology (the index of atmospheric purity and the index of damage) nearly 25 years after the initial assessment. The authors found a general decrease in lichen biodiversity in several sampling plots and a widespread increase in damage symptoms in the selected lichen species studied in 1997 compared with those from 2022. Although these changes appear to be the result of a multifactorial response, air pollution impacts and climate change were pointed out as major players. Further, this study supports the usefulness of the near-infrared molecular fingerprint together with advanced statistical methods for assessing the effects of global change drivers in the lichen community biological traits assessment. 

Studies addressing the effects of the interaction between different global change drivers on ecosystems are scarce, and extremely rare in the field of lichens. Morillas et al. [11] analyzed the interactive effect of climate change and atmospheric nitrogen deposition on a soil biocrust-forming lichen in Lisbon. In this study, the authors investigated the impacts of different long-term water availability regimes simulating climate changes and their interaction with nitrogen additions on the physiological response of the soil lichen *Cladonia rangiferina*. The results showed that the interaction between reduced watering and an increased nitrogen supply, combined with temperature changes, can compromise the physiological performance of this soil lichen. Contrary to the conventional belief that increased nitrogen levels would impact ecophysiological responses in soil lichens, potentially even in a linear manner, this study demonstrates that the actual behavior is dependent on interactions with other global change drivers. 

Another important facet of research in the field of lichens regards genomic resources, which serve as critical tools that inform conservation efforts aiming to protect threatened species. Leavitt et al. [12] analyzed the reference genomes of the endangered soil lichen species Florida Perforate Cladonia within the Florida scrub in the southeastern USA. The authors used high-throughput sequencing to analyze the reference genomes, which play a crucial role in understanding the genomic diversity, local adaptations and landscape-level genetics of this species. Through genome-scale data, they assembled the first draft nuclear and mitochondrial genomes for the mycobiont (*Cladonia perforate*) and they assessed the genetic diversity of the species within and among populations in southeastern Florida. The analysis revealed the presence of multiple distinct mycobiont parental genotypes at local scales in southeastern Florida. They concluded that the advantages of translocating individuals from robust populations to restore disturbed populations surpass the risk associated with introducing novel genetic variation.

Because the distribution of lichen species is highly correlated with environmental conditions such as air pollution and climate, the study of distribution maps of lichen bioindicators can provide information on trends in environmental factors contributing to predictions of future ecosystem changes. Farkas et al. [13] assessed the lichen distribution data from herbarium collections, recent field studies and the literature on lichens growing on different surfaces (saxicolous, terricolous, corticolous, and lignicolous lichens) throughout Hungary. To categorize the different distribution types, the authors identified five categories, depending on the evolution of the species occurrence in recent years or decades. The study emphasizes the value of such analyses for bioindication, providing insights into current and future climatic and pollution situations. Furthermore, the authors propose using the categories of distribution types of the lichens used in this study as potential tools to extrapolate this kind of analysis to wider regions such as the European or global level.

Overall, this Special Issue highlights the relevance of accounting for the effects of long-term studies and interactions among environmental factors in studies applying lichens as bioindicators of global change drivers. Further, the Special Issue supports the importance of using lichen genomic resources as conservation tools, as well as studying the distribution maps of lichen bioindicators to project future climatic and pollution scenarios. Lastly, it has underscored the need to protect the natural diversity of tree species as epiphytic lichen hosts. Overall, this Special Issue covers many, although not all, aspects of the role of lichens as bioindicators of global change drivers. Future research areas in this field must delve into the need to reveal how interactions between global change drivers affect morphological, physiological and metabolic responses of lichens, as such interactions could trigger complex responses, which are unpredictable from one-factor studies [14]. However, studies covering the effects of multiple global change drivers on lichens responses are extremely scarce. Another pressing issue within this field is to reveal not only the response of the dominant mycobiont to the environmental changes, but also the response of the vast array of related microorganisms that form its microbiome [15,16]. The potential different roles of the various organisms involved in this complex holobiont should be explored in the context of global changes to obtain a deep understanding of the functioning of these organisms. 
